# The c.1A > C start codon mutation in *CLN3* is associated with a protracted disease course

**DOI:** 10.1002/jmd2.12097

**Published:** 2020-02-07

**Authors:** Willemijn F. E. Kuper, Claudia van Alfen, Linda van Eck, Stella A. de Man, Marjolein H. Willemsen, Koen L. I. van Gassen, Monique Losekoot, Peter M. van Hasselt

**Affiliations:** ^1^ Department of Metabolic Diseases, Wilhelmina Children's Hospital University Medical Center Utrecht, Utrecht University Utrecht The Netherlands; ^2^ Bartiméus Institute for the Visually Impaired Zeist, Doorn The Netherlands; ^3^ Department of Pediatrics Amphia Hospital Breda The Netherlands; ^4^ Department of Human Genetics Radboud University Medical Center Nijmegen The Netherlands; ^5^ Department of Genetics University Medical Center Utrecht Utrecht The Netherlands; ^6^ Department of Clinical Genetics Leiden University Medical Center Leiden The Netherlands

**Keywords:** CLN3, counseling, protracted phenotype, start codon, translation initiation codon

## Abstract

**Background:**

CLN3 disease is a disorder of lysosomal homeostasis predominantly affecting the retina and the brain. The severity of the underlying mutations in *CLN3* particularly determines onset and course of neurological deterioration. Given the highly conserved start codon code among eukaryotic species, we expected a variant in the start codon of *CLN3* to give rise to the classical, that is, severe, phenotype.

**Case series:**

We present three patients with an identical *CLN3* genotype (compound heterozygosity for the common 1 kb deletion in combination with a c.1A > C start codon variant) who all displayed a more attenuated phenotype than expected. While their retinal phenotype was similar to as expected in classical CLN3 disease, their neurological phenotype was delayed. Two patients had an early onset of cognitive impairment, but a particularly slow deterioration afterwards without any obvious motor impairment. The third patient also had a late onset of cognitive impairment.

**Conclusions:**

Contrasting our initial expectations, patients with a start codon variant in *CLN3* may display a protracted phenotype. Future work will have to reveal the exact mechanism behind the assumed residual protein synthesis, and determine whether this may be eligible to start codon targeted therapy.

## 1 INTRODUCTION

The start codon is responsible for the initiation of transcription and errors herein are intuitively expected to result in the absence of a functional protein.^1^ As phenotype correlates with genotype, a severe phenotype is expected. In our clinic, we identified a patient with the common 1 kb deletion and a c.1A > C start codon variant in *CLN3*.^2^ CLN3 disease (OMIM #204200) is a neurodegenerative lysosomal storage disorder predominantly affecting the retina and the brain. In the classical phenotype—deriving from two truncating mutations expected to completely abolish CLN3 protein synthesis—neurodegeneration already starts and progresses in childhood limiting lifespan of patients to their late teens/early 20s.^2^, ^3^, ^4^ Contrary to our expectations, the patient displayed a protracted phenotype—defined as deriving from at least one “mild” (missense) mutation still allowing some residual protein synthesis associated with a delayed neurological phenotype.^2^ The milder phenotype could not be explained by the 1 kb deletion on the other allele, as this common founder mutation severely affects CLN3 protein synthesis generally hypothesized to completely abolish CLN3 protein function.^5^, ^6^ Indeed, although some degree of phenotypic heterogeneity is seen, homozygosity for the 1 kb deletion—present in around 75% of CLN3 disease patients—is consequently associated with the classical phenotype.^2^, ^5^, ^7^, ^8^

In this report, we assessed the hypothesis that the c.1A > C variant in *CLN3* is responsible for a protracted CLN3 disease phenotype by successfully searching for additional patients harboring this genotype and delineating their disease course.

## 2 CASE SERIES

### 2.1 Case 1

**Figure 1:**
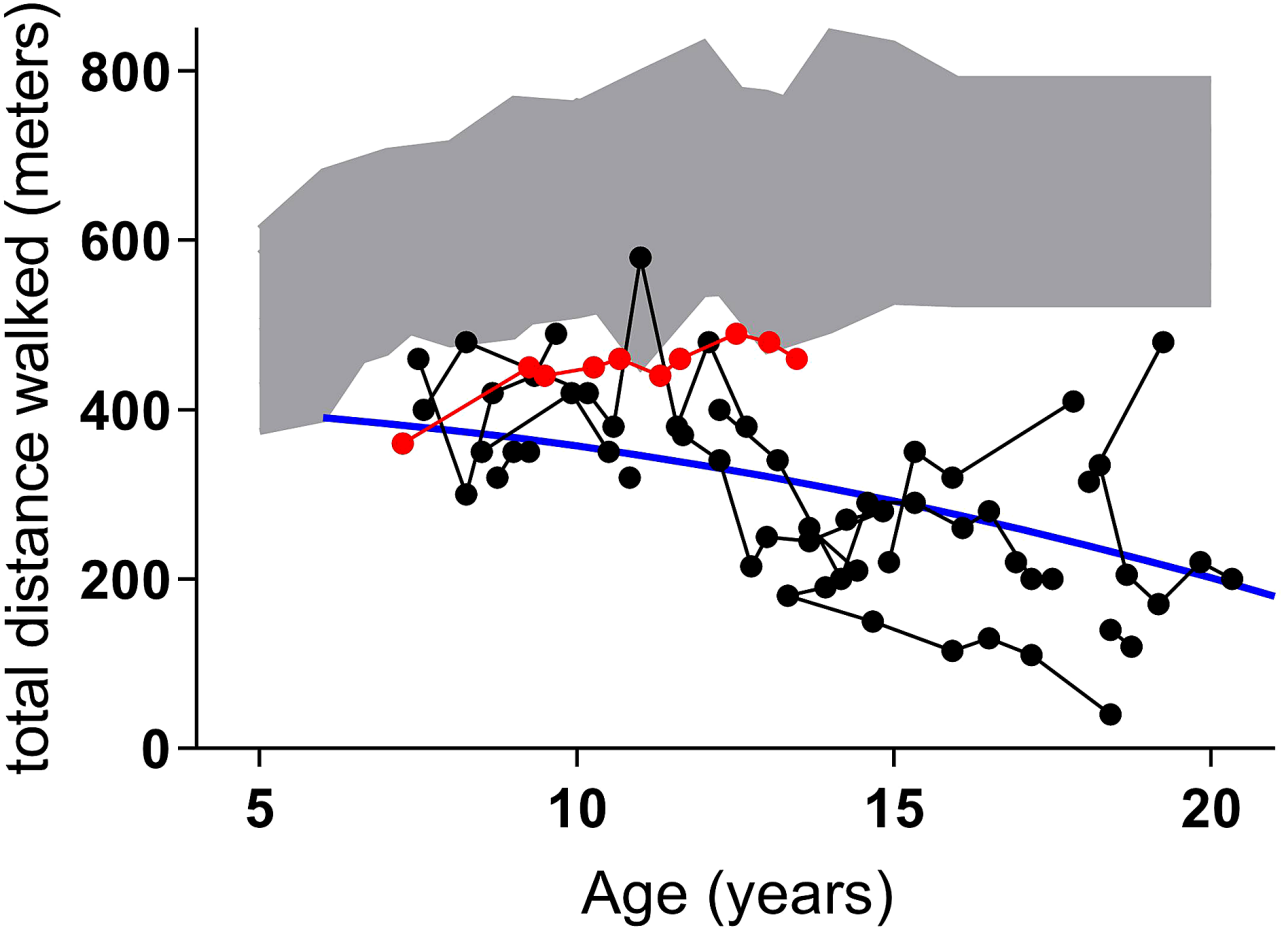
Six‐minute walk test (6MWT) performance in a patient with the 1.A > C start codon mutation in *CLN3* compared to classical CLN3 disease. Figure adjusted from Kuper et al.^4^ The red dots show the 6MWT performance of case 1 indicating a stable, near‐normal, walking distance. This increasingly deviates from the classical CLN3 disease decline as depicted by the black dots (individual test results) and the blue line (overall velocity of the 6MWT distance decline over time). The grey area shows the 6MWT reference values in healthy, sighted children (+/−2 SD)

This 14‐year‐old boy is the only child born to unrelated, Dutch parents. In his early childhood, a mild developmental delay was noticed, particularly concerning behavioral abnormalities, which led to the diagnosis of an autism spectrum disorder. Around the age of 5 years, a rapid decrease of vision was noticed and following ophthalmological examinations a cone‐rod dystrophy was identified. Due to the combination of a retinal dystrophy with a mild developmental delay, the suspicion of CLN3 disease was raised. At the age of 6 years, the diagnosis of CLN3 disease was confirmed based on compound heterozygosity for the 1 kb deletion and a c.1A > C start codon variant in *CLN3*. Ever since presentation, the patient has remained essentially stable, without clear signs of cognitive or motor deterioration. Regarding cognitive functions, his total IQ (TIQ) of 71 around diagnosis at 6 years of age has only declined to a verbal IQ (VIQ) of 67 at 12 years of age. Although TIQ and VIQ (at 12 years a TIQ could no longer be assessed since the patient is completely blind) are not directly interchangeable, this does suggest some degree of cognitive stabilization. More clearly, regarding motor function, this patient has walked a near‐normal, stable 6‐minute walk test distance in the past years ‐ contrasting the early and continuous decline seen in (classical) CLN3 disease^4^ (Figure 1). Recently, at the age of 13 years, he has experienced his first generalized tonic‐clonic seizure.

### 2.2 Case 2

This woman, currently in her mid‐twenties, was born to unrelated, Dutch parents. Her younger sister and brother are healthy. Of note, two far relatives had died due to CLN3 disease. No further details, particularly no genetic information, could be retrieved. Her early development was reportedly normal. Around the age of 7 years a rapid decrease in her vision was noticed. A retinal dystrophy affecting the macula was found, which, in combination with the suggested family history of CLN3 disease, led to the identification of two compound heterozygous disease causing variants in *CLN3* (the 1 kb deletion + c.1A > C start codon variant). Following the diagnosis of CLN3 disease, her parents were counseled on the expected disease course in classical CLN3 disease. They therefore expected that she would decline severely in the following years resulting in premature death around 20 years of age. In the following years however, psychomotor deterioration was profoundly slower than expected. While she currently, at the age of 24, has exhibited some degree of deterioration, the deterioration is limited mainly concerning short term memory, concentrating on multiple tasks and word finding. She is still able to perform simple tasks on a farm specialized in care for the mentally handicapped. Similarly, and perhaps even more strikingly, her motor function still seems preserved. She does not experience any extrapyramidal symptoms: her speech is still clear and she still walks independently without the need for a wheelchair. Only since the age of 23 years, she has started to suffer from generalized tonic‐clonic seizures.

### 2.3 Case 3

This woman was the first child born to unrelated, Dutch parents with a healthy younger sister. Following a normal early development, a rapid loss of vision was noticed at the age of 7 years initially attributed to juvenile macular degeneration (Stargardt disease). However, after her first generalized tonic‐clonic seizure at 11 years of age she was diagnosed with CLN3 disease based on a skin biopsy and genetic analysis, although only one disease causing variant (the common 1 kb deletion) could be identified at that time. Despite the expected deterioration, her cognitive and motoric abilities remained preserved for years. With practical adjustments for her visual handicap, she was able to follow regular level education and even to graduate from high school at the age of 18 years which has, to our knowledge, not been reported in classical CLN3 disease and which strongly implies an (above) average IQ until her late teens. Around her final year at high school, however, a decrease in her learning abilities and short term memory became increasingly obvious. In the following years, she quickly deteriorated both in her cognitive and motoric abilities. From her early twenties onwards, she lost the ability to walk and her speech became unintelligible. She died just before the age of 26 years. After she passed away, carrier ship analysis within the family led to the identification of the second variant—the c.1A > C start codon variant—in *CLN3*.

## 3 DISCUSSION

We report on three CLN3 disease patients compound heterozygous for the common 1 kb deletion and a c.1A > C start codon variant in *CLN3* who all displayed a protracted phenotype. The CLN3 protein is a transmembrane protein with yet unresolved function. In the absence of a specific antibody (precluding reliable residual protein analysis) and/or an assay measuring residual activity we focused on the clinical underpinning.^9^

Based on this milder clinical presentation, we hypothesize that the c.1A > C variant in *CLN3* does not completely abolish CLN3 protein synthesis. Interestingly, although start codon variants are often associated with a severe disease course,^10^, ^11^ there are several reports of disease causing start codon variants describing a milder phenotype.^12^, ^13^, ^14^ The residual protein synthesis in case of such a start codon variant may be explained by the production of a shorter but still partly functional protein because of the use of an alternative downstream start codon, either in the mutated transcript itself or in alternatively spliced transcripts. The CLN3 protein is known to exist in multiple isoforms, of which a few have a putative later start position—recently extensively reviewed by Mirza et al^6^—and thus do not encompass the mutated c.1A > C base pair as present in our patients. Possibly, these unaffected isoforms may provide the residual CLN3 protein production allowing a protracted neurological phenotype. An alternative hypothesis is that the mutated start codon itself is still translated. From in vitro studies, non‐AUG translation initiation has been shown to occur in a wide range of proteins, with some proteins even deriving solely from a non‐AUG start codon. Since non‐AUG translation initiation occurs at a much lower efficiency than the regular AUG translation initiation, this is probably one of the processes to regulate protein expression. Of the non‐AUG start codons, the CUG alternative start codon—which corresponds to the c.1A > C start codon variant in our patients—has been suggested to provide the “least inefficient” start codon.^15^ However, whether this tightly controlled regulatory process also applies to a variant in the start codon is speculative. In addition to transcriptional (regulatory) processes as described above, also, other—genetic, such as post‐translational modifications, and probably non‐genetic—modifying factors could impact residual CLN3 protein synthesis and function.^6^ Regardless of the exact underlying mechanism, the protracted phenotype displayed by all three patients discussed in this report strongly indicates that the c.1A > C start codon variant in *CLN3* allows some degree of residual protein synthesis.

Even with the start codon mutation being responsible for some degree of residual protein synthesis, it remains particularly puzzling why two of our patients displayed early onset of cognitive impairment, but thereafter a particularly slow decline/long plateau phase contrasting case 3 who finished regular high school but thereafter declined dramatically rapidly. Regarding the first two patients, particularly in light of (near) future therapeutic efficacy studies, prudence is warranted when classifying patients with an atypical genotype as having classical CLN3 disease, even if confirmed by an impaired IQ and motor score (as in case 1) as they might still later display a protracted phenotype. Regarding the third patient, insight in the chain of events that led the “neuronal” balance to collapse so dramatically might also provide insight in neuronal deterioration in CLN3 disease in general.

In humans, previous work has shed light on the factors that determine whether a start codon variant will cause disease.^16^ Our work suggests that future work should focus on the next step: the factors that determine whether a disease causing start codon variant completely abolishes protein synthesis or still allows some residual protein synthesis and the mechanism behind this. Insight herein will improve diagnosis, counseling, follow‐up and possibly even therapy development analogous to the concept of nonsense codon read through.^17^, ^18^

## CONFLICT OF INTEREST

The authors declare no potential conflict of interest.

## AUTHOR CONTRIBUTIONS

W.F.E.K.: study design, data acquisition, interpretation of data, and drafting of manuscript. C.A.: data acquisition, interpretation of data, and critical revision of the manuscript for intellectual content. L.E.: data acquisition, interpretation of data, and critical revision of the manuscript for intellectual content. S.A.M.: data acquisition, interpretation of data, and critical revision of the manuscript for intellectual content. M.H.W.: data acquisition, interpretation of data, and critical revision of the manuscript for intellectual content. K.L.I.G.: interpretation of data and critical revision of the manuscript for intellectual content. M.L.: data acquisition, interpretation of data, and critical revision of the manuscript for intellectual content. P.M.H.: study design, interpretation of data, and critical revision of the manuscript for intellectual content.

## ETHICS STATEMENT

Written consent to publish the case series was provided by the parents/legal representatives of the patients.

## Data Availability

Anonymized data will be shared by request from any qualified investigator.
